# Alternative splicing plays key roles in response to stress across different stages of fighting in the fish *Betta splendens*

**DOI:** 10.1186/s12864-022-08609-2

**Published:** 2022-05-30

**Authors:** Vu Trieu-Duc, Kenshiro Oshima, Kenya Matsumura, Yuri Iwasaki, Ming-Tzu Chiu, Masato Nikaido, Norihiro Okada

**Affiliations:** 1 School of Pharmacy, Kitasato University, Tokyo, Japan; 2 Life Sciences and Biotechnology Department, Tokyo Institute of Technology, Tokyo, Japan; 3 Department of Life Sciences, National Cheng Kung University, Tainan, Taiwan; 4 Nagahama Institute of Bio-Science and Technology, Nagahama, Japan

**Keywords:** Alternative splicing, *B. splendens*, Stress, RNA-seq, Fighting interaction, Transcriptome

## Abstract

**Background:**

Aggression is an evolutionarily conserved behavior critical for animal survival. In the fish *Betta splendens*, across different stages of fighting interactions, fighting opponents suffer from various stressors, especially from the great demand for oxygen. Using RNA sequencing, we profiled differential alternative splicing (DAS) events in the brains of fish collected before fighting, during fighting, and after fighting to study the involvement of alternative splicing (AS) in the response to stress during the fight.

**Results:**

We found that fighting interactions induced the greatest increase in AS in the ‘during-fighting’ fish, followed by that of the ‘after-fighting’ fish. Intron retention (IR) was the most enriched type among all the basic AS events. DAS genes were mainly associated with synapse assembly, ion transport, and regulation of protein secretion. We further observed that IR events significantly differentiated between winners and losers for 19 genes, which were associated with messenger RNA biogenesis, DNA repair, and transcription machinery. These genes share many common features, including shorter intron length and higher GC content.

**Conclusions:**

This study is the first comprehensive view of AS induced by fighting interactions in a fish species across different stages of those interactions, especially with respect to IR events in winners and losers. Together, these findings facilitate future investigations into transcriptome complexity and AS regulation in response to stress under the context of aggression in vertebrates.

**Supplementary Information:**

The online version contains supplementary material available at 10.1186/s12864-022-08609-2.

## Background

Aggression is an evolutionarily conserved behavior critical for animal survival [1]. Using aggressive behavior, animals of many species establish dominance hierarchies that can be observed both in nature and in the laboratory [2]. Ranks in these hierarchies can induce stress that directly affects many aspects of the lives of these animals, including their physiology, genetic expression, and neurogenesis [3]. Animals need to respond to this stress rapidly and effectively at the physiological, cellular, and molecular level to increase their chance of survival.

Thanks to the development of high-throughput sequencing technologies such as RNA sequencing (RNA-seq), transcriptomic analyses have become useful for studies of aggression at the level of gene expression, as exemplified by those of differentially expressed genes (DEGs) in dominant and subordinate cichlid fish [4], in zebrafish [5], and in stickleback fish [6]. These studies have consisted of mainly analyses of DEGs under different aggressive conditions and have revealed various genes that are involved primarily in oxidative stress, ion transport, and energy metabolism pathways [7]. Currently, few studies have focused on post-transcriptional regulation in response to stress in fighting opponents, although the importance of alternative splicing (AS) in the context of social dominance has long been accepted [8].

AS, the process by which structurally and functionally distinct mRNAs are generated from a single gene, which ultimately leads to protein variants, has key roles in enhancing regulatory capacities and proteomic complexities in eukaryotes [9]. For example, it is estimated that 90% of the genes in the human genome are spliced in alternative ways to generate more than one protein per gene [10]. In the rat, *Slo*, which encodes a potassium channel expressed in neurons, has the potential to encode 500 alternative versions of that product [11]. Remarkably, *Dscam*, a *Drosophila* exon guidance receptor gene, can encode as many as 38,000 possible products as a result of AS [12]. These genes are implicated in nervous system functions, suggesting a crucial role for AS in establishing the highly complex responses of animal neurons.

AS represents a tightly regulated response to various stresses that leads to transcriptome plasticity. It is a key player in response to heat stress in catfish [13], hypoxia stress in Nile tilapia [14], and salinity stress in spotted sea bass fish [15] and has been associated with neurogenesis [16]. Five basic types of AS have been extensively studied, including exon skipping (ES), alternative 5’ splice site (A5SS), alternative 3’ splice site (A3SS), intron retention (IR), and mutually exclusive exon (MXE) [17]. Of these AS types, IR has been reported to have an important regulatory role in response to hypoxia stress [18], regulation of mRNA expression patterns during hematopoiesis [19], and neurogenesis [20]. However, there have been few studies on stress-related AS in general and on that in fish within the context of aggression in particular.

The fighting fish *Betta splendens* is an anabantoid from Southeast Asia. In nature, males defend territories in the water column near the surface. This species is very aggressive and has very stereotypical social displays, leading to its wide use in laboratory studies of aggressive interactions. The social displays of *B. splendens* have been described in detail [7, 21]. Fighting interactions between opponents can last for more than an hour. A long fight duration is dangerous and often induces various sources of stress and can even result in the death of one of the opponents. Therefore, we would expect to see the involvement of AS in response to stress across different stages of the fighting interaction in this fish, especially in the winners and losers of the fight.

To this end, using RNA-seq datasets, we identified and characterized AS profiles and differential alternative splicing (DAS) events and DAS genes in *B. splendens* under different fighting durations, namely non-fighting, during fighting, and after fighting. We identified several DAS events across different fighting durations, among which IR events were the most common in all fighting stages. Several genes that showed differential IR between winners and losers were associated with messenger RNA biogenesis, mitochondrial biogenesis, and DNA repair. Our findings will be helpful for understanding the stress-related AS mechanism in vertebrates.

## Results

### Overview of AS events in *B. splendens*

**Fig. 1:**
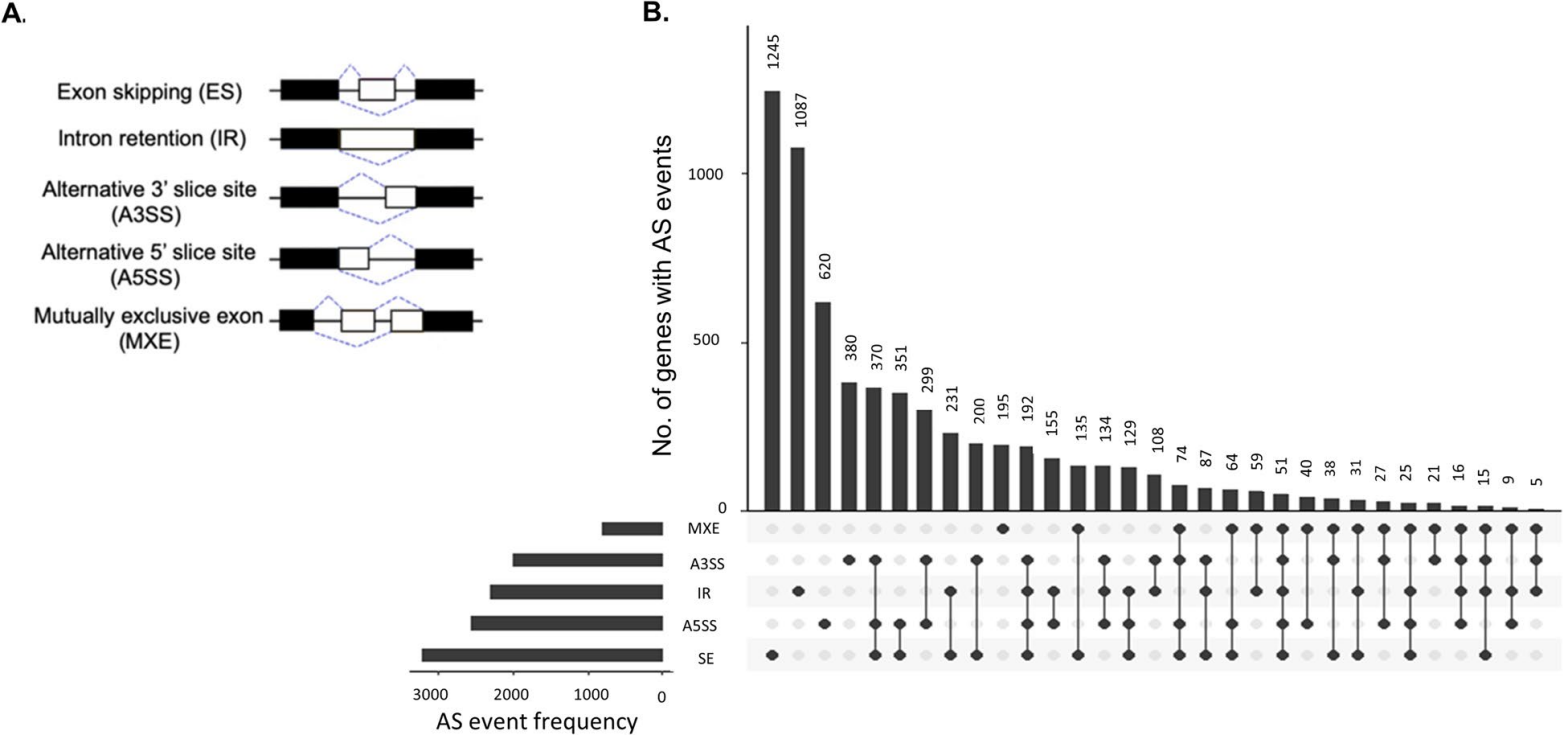
UpSet plot of interactions between five types of AS events and involved genes. **A** Illustration of give classical AS events. **B** In the upper part, a bar plot shows the number of genes with AS events. In the lower part, each row represents an AS event type with the black circles highlighting the observed AS events and black lines connecting those observed AS events generated from a particular gene set in the upper bar

First, we used ASTALAVISTA to determine the AS types based on the genomic information and RNA-seq samples pooled from 37 brain samples. A total of 46,865 AS events generated from 16,691 genes were identified in male *B. splendens*. According to different splicing patterns, these AS events can be divided into six types: ES, IR, A3SS, A5SS, ME, and others (OT) (Fig. 1A, Additional file 1). Here we focused on five basic AS types, ES, IR, A3SS, A5SS, and ME, and observed that a total of 15,442 AS events belonged to these types and were generated from 10,577 genes. Of these 15,442 AS events, the most enriched type of AS event was A5SS, accounting for 29.53%, followed by ES (28.76%), A3SS (20.61%), RI (15.44%), and ME (5.66%) (Fig. S1).

Next, we constructed an UpSet plot to intuitively visualize the intersecting sets of each gene (among the 10,577 genes) and five AS types. The results showed that roughly 33% of the AS genes (3,518 genes) were associated with only a single type of AS event, including 1,245 SE events, 1,078 RI events, 620 A5SS events, and 195 MXE events (Fig. 1B). In contrast, the remaining genes were associated with at least two AS events, and some underwent as many as five AS events. This latter group is exemplified by the genes *calcium channel voltage-dependent* (*cacna1bb* and *cacna1ab*), which are involved in membrane depolarization during neuronal action potentials and calcium ion transport, as well as *rabep1*, *csnk1ga*, and *trip10a*, which have a role in endocytosis (see Additional file 2).

**Fig. 2:**
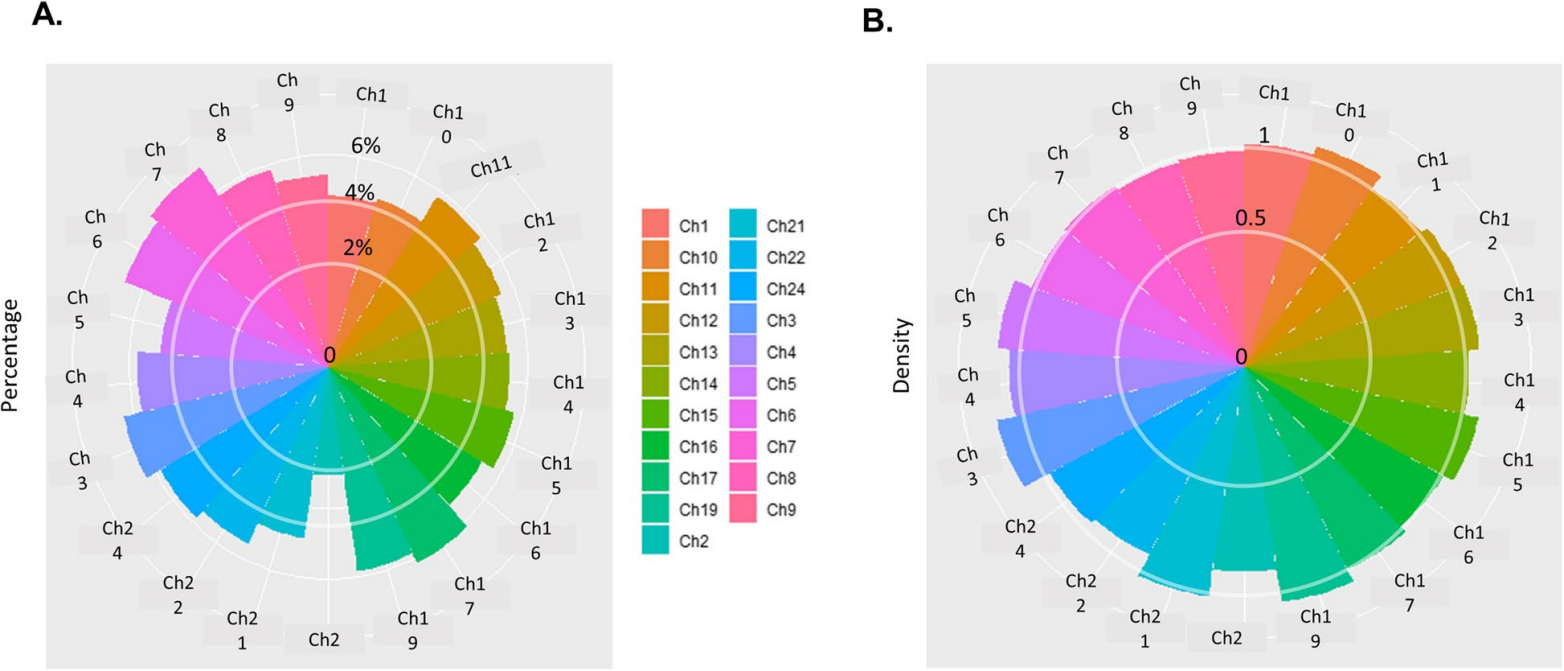
Distribution of AS events and genes in the reference genome of *B. splendens.* **A** The average percentage of genes with AS events in each chromosome; the outermost circle indicates 7%. **B** AS event density (AS event number/AS gene number) of each chromosome; the outermost circle indicates a density of 1.4. Each chromosome is represented by a unique color

Finally, we used Circos plots to investigate the distribution of AS events and genes in the reference genome of *B. splendens*. The percentage of genes with AS events and the average AS event density (AS event number/gene number) were calculated for each chromosome (Chr). We observed that the percentage of genes with AS events was roughly 5% (Fig. 2A), and among these genes ~ 5% were associated with one AS event (AS event density) (Fig. 2B).

### AS event profiles in the brains under different fighting durations

**Fig. 3:**
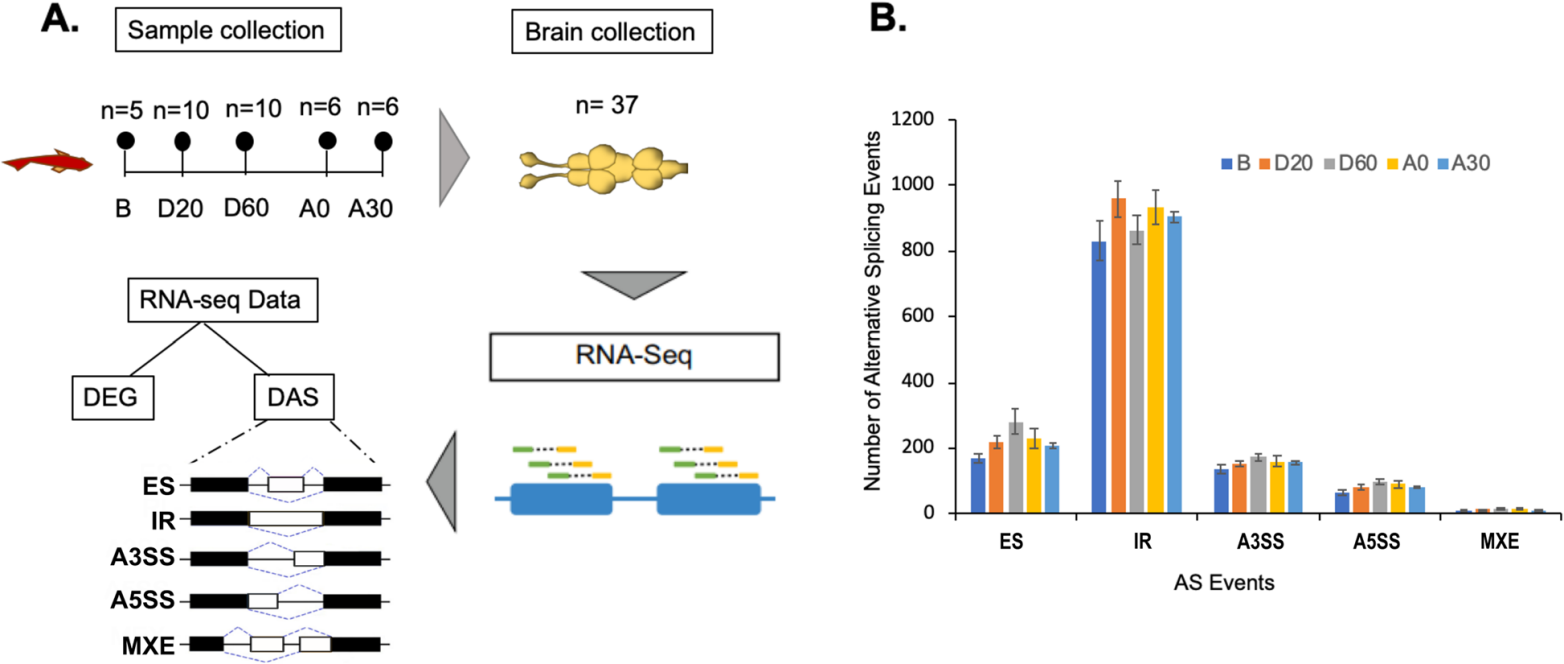
AS event profiles in *B. splendens* brains under different fighting durations. **A** Schematic illustration of the study design highlighting the fish, brain, and types of data analyses. The RNA-seq data analyses were used to determine DEGs and DAS genes. **B** The number of AS events detected in 37 brain samples (average ± SE)

We then used ASTALAVISTA to evaluate the detailed AS event profiles of brains of *B. splendens* under five fighting durations, i.e., in the absence of fighting (corresponding to the before fighting state, B), during fighting at 20 min (D20) and 60 min (D60), just after the conclusion of a fight (A0), and 30 min after the end of a fight (A30) (Fig. 3A). Of the five basic AS types, a total of 1,215; 1,424; 1,431; 1,428; and 1,356 AS events were identified in the B, D20, D60, A0, and A30 groups, respectively (Fig. 3B, Additional file 3). In brief, fighting interactions induced increases in the number of all types of AS events, which were ~ 1.2 times higher in the during-fighting individuals (D20 and D60) and the after-fighting individuals (A0 and A30) relative to the non-fighting individuals. Strikingly, the most common type of AS event across all groups was IR, which accounted for 60.3%, 68.4%, 65.2%, 67.3%, and 66.6% of AS events in the B, D20, D60, A0, and A30 group, respectively (Fig. 3B).

### DAS genes in the comparison between different fighting durations

First, DAS events between different fighting durations were determined using rMATS. Splicing was characterized based on the relative proportion of two alternative isoforms at each splice site, which is referred to as the percent spliced index (PSI). A PSI value of 1 or 0 indicates that only one of the two alternative isoforms was expressed, and a value of 0.5 indicates equal expression of both isoforms. Several DAS events were detected across all possible comparisons e.g., B vs. D20, B vs. D60, B vs. A0, etc. (Additional file 4). Here, we use the SE event, which was the most common DAS type across all comparisons, for a demonstration of PSI estimation (Fig. S3A). Using hierarchical clustering for 508 common loci (i.e., loci with at least five counts across all individuals in all fighting groups), we found that SE splicing was similar between the B and D20 groups as well as between the D60 and A0 groups, but the A30 group was clustered separately (Fig. S3B). We note, however, that there was no significant difference in the average percent PSI value for all common loci across all comparisons (Fig. S3C). This finding suggested that differential SE splicing occurred with biological significance at only specific loci.

**Fig. 4:**
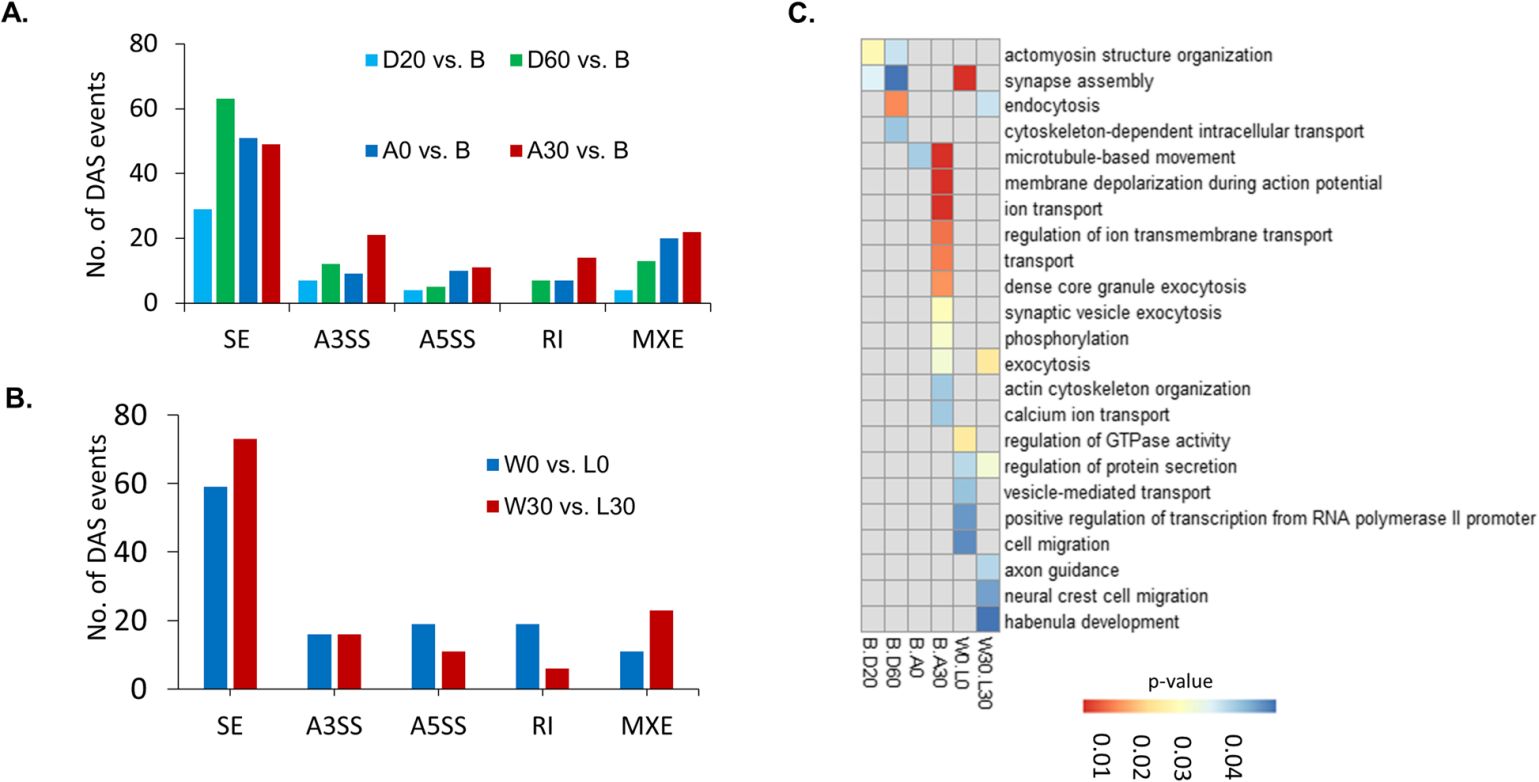
Number of DAS events detected between different comparisons. **A** DAS events detected from the comparison between fighting groups (D20, D60, A0, and A30) and the non-fighting group (**B**). **B** Number of DAS events detected from the comparison between winners and losers. **C** Enriched biological process GO terms for the DAS genes isolated from each comparison

Next, we focused on identifying the DAS events and DAS genes for the comparison between all fighting groups (D20, D60, A0, and A30) and the non-fighting group (B). We observed 44, 100, 97, and 117 DAS events in D20 vs. B, D60 vs. B, A0 vs. B, and A30 vs. B (Fig. 4A, Additional file 4), corresponding to 43, 82, 90, and 114 DAS genes, respectively. We further determined the DEGs for D20 vs. B, D60 vs. B, A0 vs. B, and A30 vs. B and identified 645, 2807, 2692, and 1526 DEGs, respectively. Then, we examined the relationships between DEGs and DAS genes by looking for the overlap between the above DEGs and the DAS gene lists and detected 0, 8, 12, and 3 DAS genes that are also DEGs in the D20 vs. B, D60 vs. B, A0 vs. B, and A30 vs. B comparisons, respectively.

In a previous study, we reported that the expression of a large number of DEGs generated from the B vs. D60 comparison was synchronized between fighting opponents of a fighting pair (Additional file 5) [7]. Here we found only five synchronized genes that were also DAS genes (*mbnl2*, *baiap2*, *snx27a*, *kif1ab*, and *ddx3xb*). In addition, we examined the DAS events, DAS genes, and DEGs between winners and losers, e.g., W0 vs. L0 and W30 vs. L30. A total of 124 DAS events (generated from 122 DAS genes) and 31 DEGs were identified for W0 vs. L0, whereas 129 DAS events (generated from 122 DAS genes) and 36 DEGs were identified for W30 vs. L30 (Fig. 4B). Noticeably, we found no genes that were both DEGs and DAS genes for either W0 vs. L0 or W30 vs. L30.

Finally, functional enrichment analysis using all DAS genes showed that DAS genes obtained from the comparisons between the during fighting groups (D20 & D60) and the B group were associated with actomyosin structure organization, endocytosis, skeleton transport and other processes. Likewise, the DAS genes generated from the comparisons between the after-fighting groups (A0 & A30) and the B group were involved in microtubule-based movement, phosphorylation, synaptic vesicle exocytosis, and other processes (Fig. 4C). Also, whereas the DAS genes for W0 vs. L0 were involved in synapse assembly, regulation of GTPase activity, and regulation of transcription from RNA polymerase II promoter, the DAS genes for W30 vs. L30 were associated with, axon guidance, neural crest cell migration, and regulation of protein secretion, (Fig. 4C).

### IR genes associated with regulation of stress in winners and losers

Accumulating evidence indicates that IR plays important regulatory roles in response to low oxygen levels, or hypoxia stress [14, 18] and in neurologic disease [22]. Thus, characterizing IR genes in W0 and L0 may allow a better understanding of how IR may regulate the hypoxia tolerance and neuronal responses in these individuals after fighting. As shown in Additional file 1, 19 genes were found to retain introns differentially between W0 and L0, among which 8 genes showed increased IR and 11 genes showed decreased IR in W0 relative to L0 (Additional file 6).

**Fig. 5:**
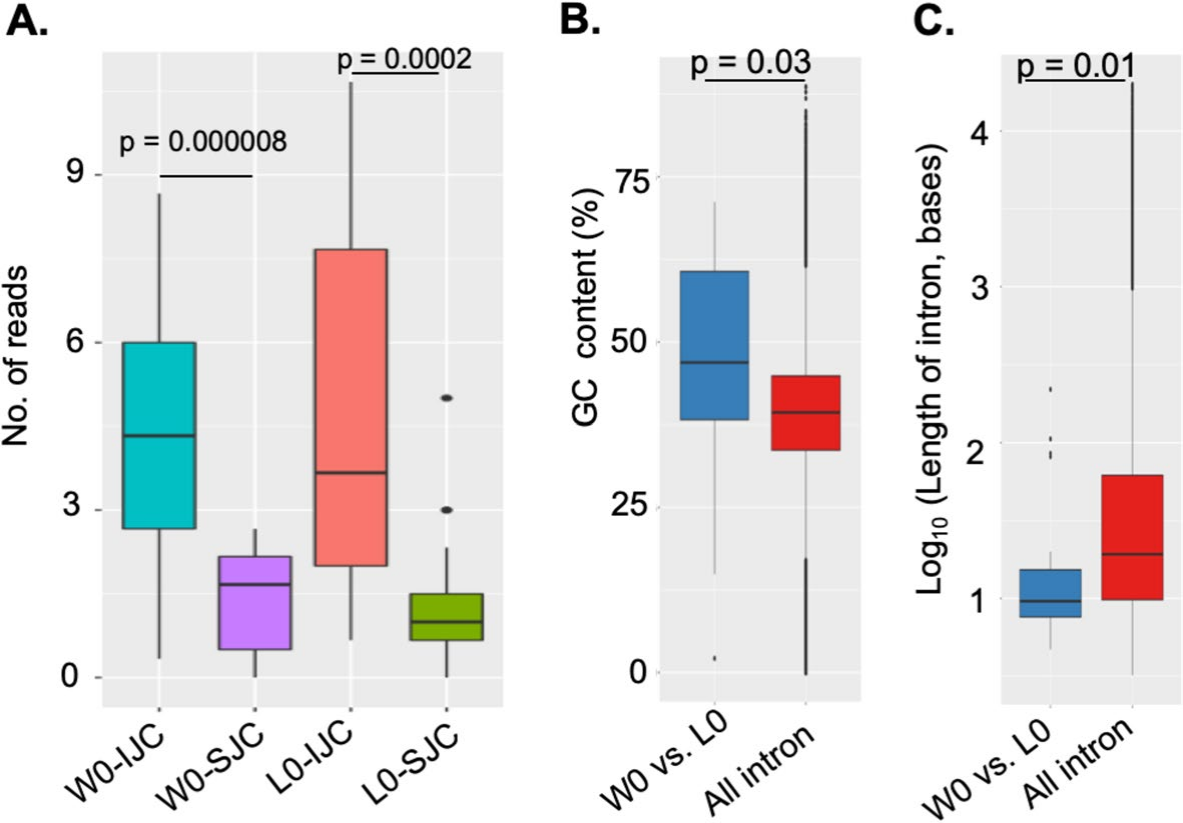
Characterization of 19 genes that underwent differential IR between W0 and L0. **A** Comparisons between IJCs and SJCs for W0 and L0. **B** Comparison of GC content among the 19 genes with retained introns and all genes with non-retained introns. **C** Comparison of intron lengths between the 19 genes with retained introns and all genes with non-retained introns. We obtained *p*-values using an unpaired t-test

Next, to analyze IR in these 19 genes mathematically, we calculated the skipping junction counts (SJCs) and inclusion junction counts (IJCs) for W0 and L0 according to the procedure described in rMATS [23] (Fig. S2). Whereas the IJCs represent the transcripts containing the intron sequence at the junction, the SJCs represent the transcripts without intron sequences at the junction. The results showed that the median value of IJCs was significantly higher than that of SJCs in both W0 and L0 (Fig. 5A), indicating that the transcripts containing introns were considerably more abundant than the transcripts without introns in these genes in both W0 and L0.

**Fig. 6:**
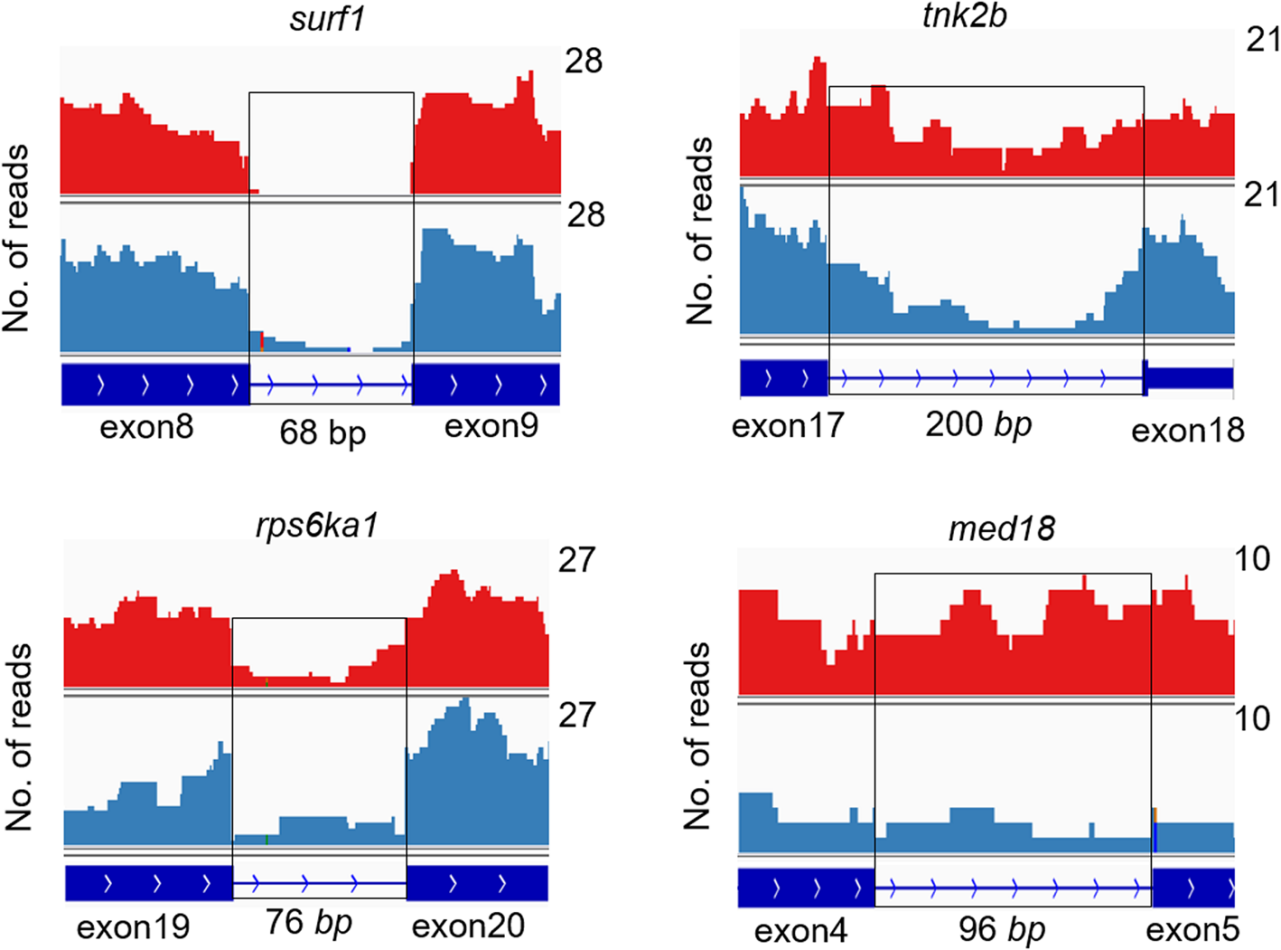
Coverage plots of four selected genes with differentially retained introns between winner and loser fish. The expression level of the retained introns and flanking exons was depicted with IGV. The retained intron is indicated inside the black box. Red shading refers to winner fish (W0), and steel blue refers to loser fish (L0). The numbers on the right side of each graph indicate the number of read counts, which were scaled to the same level between the W0 and L0 fish

Then, we examined the genomic features that are associated with retained introns in these 19 genes and found that the lengths of introns in these genes that underwent differential IR were significantly shorter (Fig. 5B) and their GC content was significantly higher (Fig. 5C) than the introns in other protein-coding genes. These data are consistent with characteristics of IR genes reported to date [24]. Among these genes was *mediator of RNA polymerase II transcription subunit 18* (*med 18*), a gene implicated in transcription machinery; *ribosomal protein S6 kinase anpha-1* (*rps6ka1*), associated with messenger RNA biogenesis; *surfeit locus protein 1* (*surf1*), associated with mitochondrial biogenesis; and *non-specific protein-tyrosine kinase* (*tnk2b*), associated with cell growth. The RIs in these genes were visualized by Integrative Genomics Viewer (IGV) (Fig. 6).

## Discussion

### AS profiles and changes in AS events during fighting in *B. splendens*

In this study, we carried out a large-scale analysis of AS profiles across different stages of fighting interactions. Our results showed that 33% of AS transcripts (3,018 genes) were generated by only single AS, whereas the remaining genes had undergone at least two events. This suggests that genes that undergo multiple AS events are widespread in *B. splendens*. This result is consistent with previous findings that environmental stresses induce increases in AS events in various fish species. For example, 492 AS events were induced in the liver of channel catfish after heat stress [13]; 103 DAS genes were identified in the heart of Nile tilapia after hypoxia stress [14]; and 502 and 162 DAS events were identified in the gill and liver, respectively, of spotted sea bass under low- vs. high-salinity conditions [15]. These findings indicate the involvement of AS events as a mechanism for the regulation of gene expression to cope with stresses in *B. splendens* within the context of aggression.

We found that AS events increased across different fighting durations, indicating that fighting interactions induce the generation of AS events in *B. splendens*, which most frequently belonged to the IR type (Fig. 3B). This tendency generally reflects our previous study showing that neuronal responses were most diverse during fighting and became stable after fighting as characterized at the level of gene expression [25]. Another previous study reported that nuclear mRNA splicing via the spliceosome is one of the most enriched biological functions for the territorial male in the case of social dominance in *Tripterygion delaisi* fish [8]. In the case of silver fox (*Vulpes vulpes*), 159 transcripts underwent DAS between tame and aggressive foxes in their brains [26]. Together, it is likely that the generation of more AS events could be a general regulatory mechanism in response to the stress of fighting interactions in fish. Apart from hypoxia stress, fighting opponents may encounter other stressors during a fight. Further studies are required to examine the causal relationship between particular AS types and stress types within the context of fighting.

### Classification of DAS genes

DAS genes in brains of *B. splendens* that have undergone different fighting durations were obtained using rMATS from various comparisons e.g., during vs. non-fighting, after vs. non-fighting, and winners vs. losers. Here, we detected 329 DAS genes from the comparisons between fighting groups (D20, D60, A0, A30) and the non-fighting group (B) and 122 DAS genes for W0 vs. L0 as well as W30 vs. L30. The Gene Ontology (GO) analyses showed that these DAS genes were associated with synapse assembly, axon guidance, calcium ion transport, and other processes (Fig. 4C). These results are supported by several lines of evidence showing that genes associated with exon guidance, synaptic connectivity, and synaptic transmission are required for rapid changes in the cellular differentiation in the nervous system [27]. However, no GO term that was shared across all fighting groups was found.

Based on the GO analyses, the DAS genes generated from the fighting groups (D20, D60, A0, and A30) vs. the non-fighting fish were generally classified into three groups. Group 1 includes the genes associated with metabolic pathways (*agpat4*, *pfkfb3*, *pdha1b*, *fad52*, *kyat1*). Among these, glycolysis (enriched in A0) is an oxygen-independent metabolic pathway that breaks down glucose into two molecules of pyruvate and produces ATP during hypoxia stress [28]. Fighting is costly because energy consumption by fighting opponents is globally increased and ATP is considerably decreased [29]; therefore, the genes enriched in the metabolic process and energy-related processes were expected. Group 2 consists of genes involved in transcription regulation such as genes associated with DNA repair and recombination (e.g., *ppp6r3*) [30], genes associated with transcription machinery (e.g., *med12*) found in D20 [31], and genes associated with RNA binding (e.g., *ddx3xb*) found in D60. Group 3 genes include those involved in post-transcriptional regulation such as *sf3a3* (found in D60), which is associated with the regulation of spliceosome assembly [32], and *ints1* (found in A30) [33] or genes associated with RNA transport such as *eif4e2* (found in D60 and A0) as well as *eif4bb* (found in A0) [34]. Similarly, we also found that some of the DAS genes isolated from W0 vs. L0 were associated with metabolism (*b4galt7*, *pigl*, and *uqcrc2b*), infection (*dync1i2b*, *kif5c*, and *vps41*), and calcium signaling (*cacna1ab*, *cacna1da*, and *phkb*); some were involved in transcription regulation (*nup210* and *upf1*); and another was involved in post-transcriptional regulation (*prpf3*).

### Relationship between DEGs, DAS genes, and IR genes

Surprisingly, there were very few genes obtained from the comparisons between fighting groups and non-fighting group that were both DEGs and DAS genes (~ 8.4%). This shows that there are cases in which species of splicing variants of a certain gene varied considerably (i.e., it had undergone DAS), but its total number of transcripts was almost the same (i.e., it was not a DEG). This was exemplified by a previous study showing that a very small proportion (19%) of overlap between DEGs and DAS genes was identified in sex determination events in embryonic day 11 mice [35]. Another good example for this was reported in the case of *rap1gap*, *a GTPase activating protein*, in which differentially spliced isoform transcript variants encode distinct proteins that lead to different functions [36]. This gene would have been overlooked as not being differentially expressed if only expression at the gene level had been considered. Thus, several isoforms produced by AS could be functionally important, although their roles have been underestimated. In future studies to identify DEGs in parallel with estimating AS events, it will be necessary for researchers to understand the complexity of the transcriptome and its functional significance.

Interestingly, there was enrichment of different GO terms between DEGs (31 genes) and DAS genes (19 genes) generated from a comparison of W0 and L0. The term ‘regulation of transcription’ was significantly enriched only for DEGs, but terms related to synaptic function, phosphorylation, and protein secretion were significantly enriched for DAS genes (Fig. 4C). The findings provide evidence that DEGs and DAS genes were involved in different biological processes in W0 and L0. These results are in accordance with observations in Nile tilapia after hypoxia stress, in which glycolysis and the oxidative stress process are enriched among DEGs, but structure of ribosome and RNA binding protein terms are enriched among DAS genes [14]. However, further investigation is required with much more transcriptomic data from other species of fish to confirm our findings.

Here we also showed that the frequency of IR events was highest in A30 relative to D20, D60, and A0 (Fig. 4A). Noticeably, among 122 DAS genes between W0 and L0, 19 genes underwent differential IR between W0 and L0. These genes were associated with transcription machinery, messenger RNA biogenesis, and mitochondrial biogenesis. In addition, 122 DAS genes were found in W30 vs. L30, among which four genes that are implicated in synaptic vesicle exocytosis (*ap3b2* and *rms1a*) and signal transduction (*dmxl2* and *si:dkeyp-234e.3*) and two other non-annotated genes underwent differential IR. A previous study showed that IR in the master regulator of translation initiation factor, namely EIF2B5, creates a stop codon that inhibits global translation to cope with hypoxia stress in cancer cells [18], or IR regulates gene expression programs [37]. Therefore, it is likely that IR events might occur to reduce the speed of production of proteins from mRNAs as a response to stress. We found that 13 of these genes had retained introns of lengths that are not divisible by three, therefore, frameshifts may have been introduced in these genes when those introns were retained (Additional file 7). We note, however, that in this study we have not confirmed whether the transcripts from these genes are indeed translated while retaining introns. Future studies are needed to confirm this possibility, with such techniques as western blots.

We previously showed that fighting opponents develop an energy-saving strategy after a fight by reducing gene expression to a minimum among all individuals in the A0 and A30 groups [25]. We also revealed that W30 and L30 fish are tolerant of oxygen deficiency and stress based on the enrichment of hypoxia response element (HRE) motifs in some DEGs [25]. Based on our cumulative findings, we propose that both winners and losers are tolerant of oxygen deficiency and stress given not only the enrichment of HRE motifs in some DEGs but also the enrichment of IR in some other genes that are expressed in the brains of these fish.

## Conclusions

In conclusion, we showed that DAS events and DAS genes considerably increased across different stages of fighting in *B. splendens*. The DAS genes were mainly classified into metabolism, translation regulation, and post-translation regulation categories. Several genes underwent significant differential IR between winners and losers and may be a part of the stress response mechanism. Taken together, these findings suggested that AS is an important mechanism in *B. splendens* in response to stress during a fight.

## Methods

### Sample collection

Fish collection and experimental procedures have been described previously in our studies [7, 25]. Briefly, several males of *B. splendens* (average standard length, 5.2 ± 1.1 cm) were used in this study. For the behavioral trials, each fighting pair, with individual fish distinguished by their colors, i.e., dark red vs. dark blue, was allowed to fight in a 1.7-L PVC tank (18 × 12.5 × 7.5 cm).

Specifically, five groups of fish were analyzed: (i) non-fighting fish (B; *n* = 5 individuals; B1, B2, B3, B4, B5); (ii) fighting for 20-min (D20; *n* = 5 pairs; D20-11 vs. D20-12, D20-21 vs. D20-22, D20-31 vs. D20-32, D20-41 vs. D20-42, and D20-51 vs. D20-52); (iii) fighting for 60-min (D60; *n* = 5 pairs; D60-11 vs. D60-12, D60-21 vs. D60-22, D60-31 vs. D60-32, D60-41 vs. D60-42, and D60-51 vs. D60-52); (iv) fish that were allowed to fight just until one fish chased the other (A0; *n* = 3 pairs; W0-1 vs. L0-1, W0-2 vs. L0-2, and W0-3 vs. L0-3), which usually takes > 1 h; (v) fish that were allowed to fight until one fish chased the other and then were collected 30 min later (A30; *n* = 3 pairs: W30-1 vs. L30-1, W30-2 vs. L30-2, and W30-3 vs. L30-3). We referred to the paired A0 fish as winners 0 (W0) and losers 0 (L0) and to the paired A30 fish as winners 30 (W30) and losers 30 (L30).

As for behavioral measurements, a total of 17 fighting pairs in which winners chased losers were videotaped and used for behavioral analyses (Additional file 8).

### Total RNA extraction and cDNA library construction

For tissue preparation, males for RNA-seq were collected before fighting (B), during fighting (D20 and D60), and after fighting (A0 and A30). The RNA extraction and mRNA library preparation have been described previously in our studies [7, 25]. Briefly, total RNA was isolated using TRIzol Reagent according to the manufacturer’s recommendation and was subsequently purified on columns with Quick-RNA MiniPrep (Zymo Research, USA). RNA-seq libraries were constructed using the TruSeq Stranded mRNA Library Prep kit (Illumina, USA) with proper quality controls, and the molar concentrations were normalized using a KAPA Library Quantification kit (Kapa Biosystems, USA) as described.

### RNA-seq

FASTQC was used to assess the quality of the reads [38]. Adaptor sequences were clipped from 50-bp single-end and paired-end sequences using the Cutadapt tool [39]. To remove low-quality bases or sequences, we trimmed the sequences using fastq_quality_trimmer software (parameters: -t 20 -l 30 -Q 33) and fastq_quality_filter software (parameters: -q 20 -p 80 -Q 33), both of which are included in the fastx toolkit v.0.0.14 (http://hannonlab.cshl.edu/fastx_toolkit/). We used 12.5 million reads per sample. We aligned reads to the fBetSpl5.2 assembly genome (https://www.ensembl.org/Betta_splendens/Info/Index?db=core) using TopHat version 2.1.1 [40] and Bowtie2 version 2.1.0 [41] with the default settings. The unique mapping reads (reads that matched the reference genome at only one position) were extracted using Samtools v.1.10 [42]. Uniquely mapped reads were counted by gene annotation (Ensembl version 101.52) using featureCounts v.1.6.3 [43]. The normalized expression levels of genes, represented by the trimmed mean of M-values, were generated with the edgeR package in R [44].

### Identification of AS in *B. splendens*

From BAM files of the mapping result, GTF annotation files of the actual observed transcript isoforms per sample were generated using stringtie v.2.1.3. The AS events observed in the isoforms shown in the GTF annotation files were classified into six groups, including exon skipping (ES), intron retention (IR), alternative 3´ splice sites (A3SS), alternative 5´ splice sites (A5SS), mutually exclusive exon (ME), and others (OT) using ASTALAVISTA v.4.0.1 [45].

### Identification of AS events in different fighting durations

AS analysis in brains of *B. splendens* under different fighting durations was conducted using the RNA-seq data generated from 37 samples as described above. The same method as described above was used to examine AS events, in which ASTALAVISTA v.4.0.1 was used to determine AS events of each sample. Only AS events detected in at least one replicated sample in the same group were considered stable AS events for subsequent analyses.

### Identification of DAS events and DAS genes

rMATS v.4.0.2 was used to detect and define five classical DAS event types, ES, IR, A3SS, A5SS, and ME [23]. Briefly, rMATS defines DAS events by computing and comparing the inclusion level (percent spliced index or PSI) of certain AS events between two RNA-seq datasets. We computed various comparisons; however, we focused on the comparisons between the non-fighting group (B) and fighting groups (D20, D60, A0, and A30) as well as between winners and losers (W0 vs. L0 and W30 vs. L30) for further analyses. A *p*-value of < 5% was set as criteria for DAS. DAS genes with differential AS events were also determined.

### Functional enrichment analysis

All DAS genes generated from the comparisons of all fighting groups (D20, A0, A30) vs. non-fighting group (B) as well as between W0 vs. L0 were used for GO (biological process term) analysis with the Database for Annotation, Visualization and Integrated Discovery (DAVID) v6.8 [46]. We tested for the overrepresentation of transcripts with a raw *p*-value of < 0.05 (Bayesian statistic).

### Visualization of genes that underwent differential IR

Four representative DAS genes, *tnk2b*, *surf1*, *med18*, and *rps6ka1*, were selected to illustrate intron retention in response to stress in W0 and L0 by using IGV [47].

## Supplementary Information

**Additional file 1.** List of expected alternative splicing events in brains of Betta splendens.

**Additional file 2.** List of alternative splicing genes and corresponding alternative splicing events.

**Additional file 3.** AS event profiles in brains under different fighting durations.

**Additional file 4.** DAS events generated from various comparisons by rMATS (*p* < 0.05).

**Additional file 5.** List of synchronized genes obtained from the B vs. D60 comparison (up-regulated genes), *p*-values were obtained by permutation test.

**Additional file 6.** List of 19 significant differential IR genes between W0 and L0

**Additional file 7.** List of genes that frameshifts may have been introduced when introns were retained.

**Additional file 8.** An ethogram of aggressive behaviors performed by fighting opponents during an initial 60-min fight.

**Additional file 9: Fig. S1.** Number of AS events and involved genes detected in 37 brain samples of *B. splendens*

**Additional file 10: Fig. S2.** Quantification of IR events from mRNA-seq data using rMATS. The IJCs represent the reads containing the intron sequence at the junction. The SJCs represent the reads without intron sequences at the junction.

**Additional file 11: Fig. S3.** Global patterns of ES events cross different fighting durations. **A** illustration of exon skipping event (**B**) heatmap and clustering of ES level. Percent spliced-in values (PSI) refer to the proportion of alternative isoforms at a splice site, where a PSI value of 1 or 0 indicates that only one of the two alternative isoforms at a splice site, and a value of 0.5 indicates equal expression of both isoforms. Numbers on each cluster represent the bootstrap probability values. **C** the average percent spliced-in value (PSI) of all isoforms across all fighting groups. Significance values were calculated using a Wilcoxon’s singled-rank test.

## Data Availability

All the data generated or analyzed during this study are included in this manuscript and its additional files 1, 2, 3, 4, 5, 6, 7, and 8. The RNA-seq data are accessible on DDBJ (https://www.ddbj.nig.ac.jp/index-e.html) with this ID: DRA009599.

## Abbreviations

AS: Alternative splicing
DAS: Differential alternative splicing
ES: Exon skipping
IR: Intron retention
A3SS: Alternative 3´ slice site
A5SS: Alternative 5´ slice site
MXE: Mutually exclusive exon
Chr: Chromosome
DEG: Differentially expressed gene
GO: Gene Ontology
PSI: Percent spliced index
