# Correction: Alternative splicing plays key roles in response to stress across different stages of fighting in the fish *Betta splendens*

**DOI:** 10.1186/s12864-022-08677-4

**Published:** 2022-06-23

**Authors:** Trieu-Duc Vu, Kenshiro Oshima, Kenya Matsumura, Yuki Iwasaki, Ming-Tzu Chiu, Masato Nikaido, Norihiro Okada

**Affiliations:** 1 School of Pharmacy, Kitasato University, Tokyo, Japan; 2 Life Sciences and Biotechnology Department, Tokyo Institute of Technology, Tokyo, Japan; 3 Department of Life Sciences, National Cheng Kung University, Tainan, Taiwan; 4 Nagahama Institute of Bio-Science and Technology, Nagahama, Japan

**Correction: BMC Genomics 22, 920 (2021)**

**https://doi.org/10.1186/s12864-022-08609-2**

Following publication of the original article [1], it was reported that there was an error in the author name of Yuki Iwasaki.

The incorrect author name is: Yuri Iwasaki.

The correct author name is: Yuki Iwasaki.

Furthermore, it was reported that the Given and Family names of Trieu-Duc Vu were transposed in the original publication.

The author group has been updated above and the original article [1] has been corrected.
