# Supplementary figures and images for: Alternative splicing plays key roles in response to stress across different stages of fighting in the fish Betta splendens
Source: BMC Genomics. 2022 May 30;22(Suppl 5):920. doi: 10.1186/s12864-022-08609-2 (PMC9150285; doi:10.1186/s12864-022-08609-2)

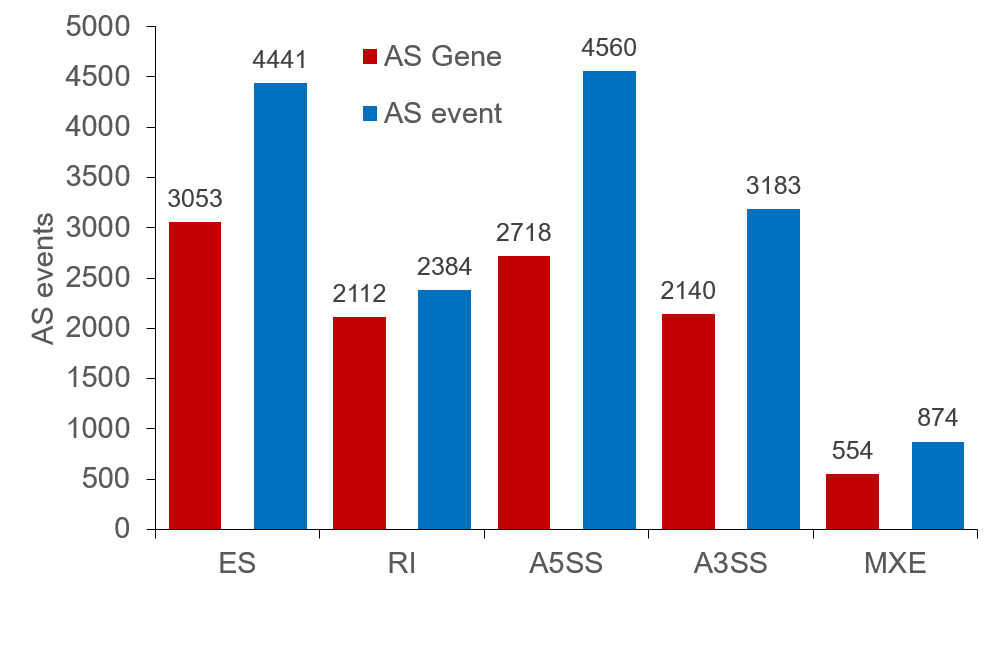

Supplement: Supplementary file 9 — Additional file 9: Fig. S1. Number of AS events and involved genes detected in 37 brain samples of B. splendens [file 12864_2022_8609_MOESM9_ESM.png]

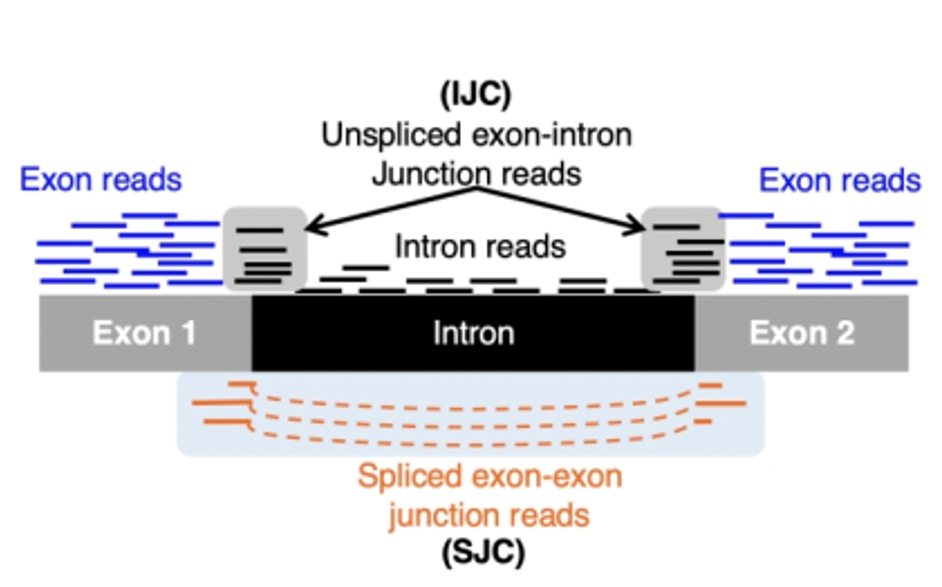

Supplement: Supplementary file 10 — Additional file 10: Fig. S2. Quantification of IR events from mRNA-seq data using rMATS. The IJCs represent the reads containing the intron sequence at the junction. The SJCs represent the reads without intron sequences at the junction. [file 12864_2022_8609_MOESM10_ESM.png]

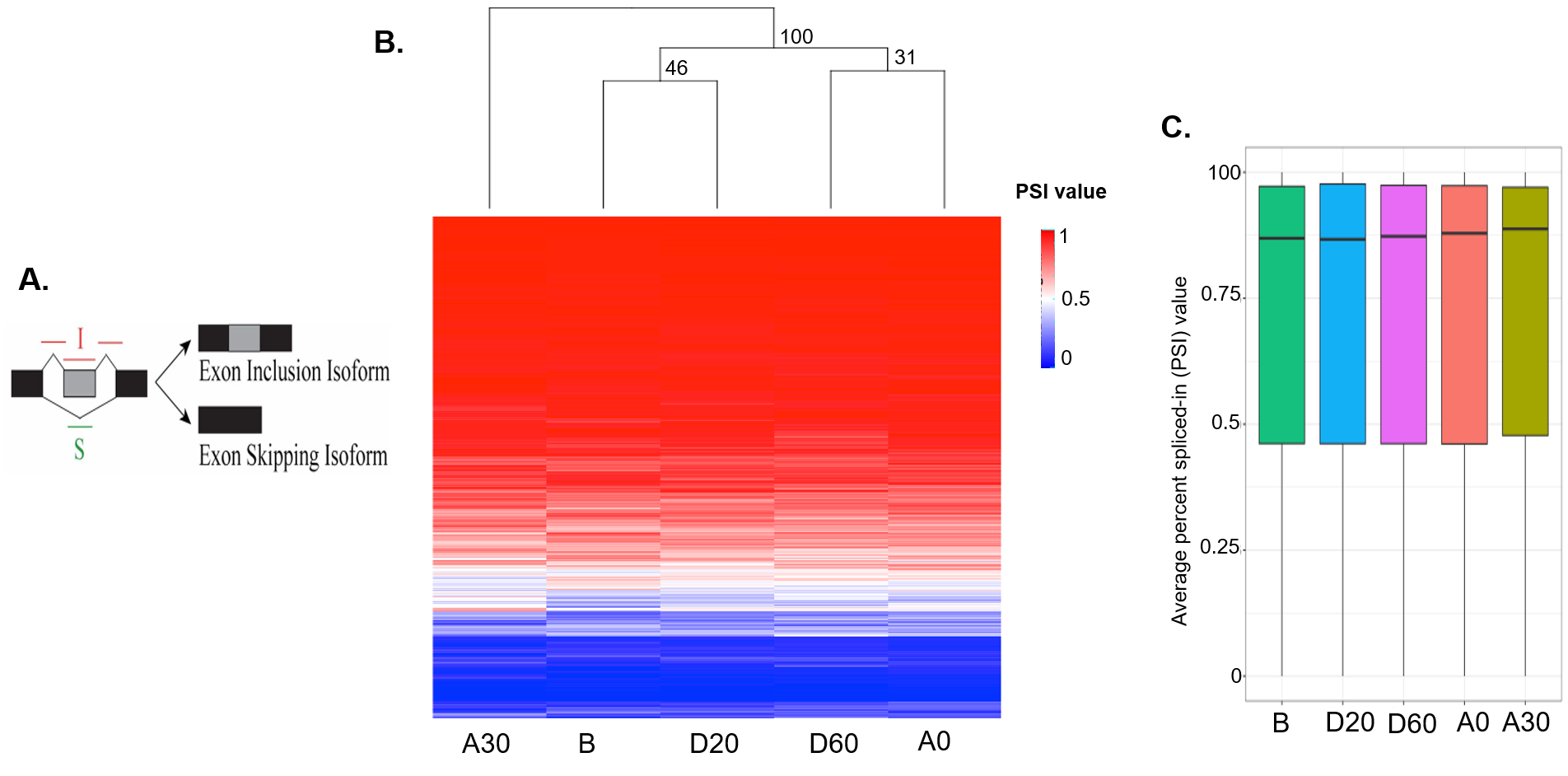

Supplement: Supplementary file 11 — Additional file 11: Fig. S3. Global patterns of ES events cross different fighting durations. A illustration of exon skipping event (B) heatmap and clustering of ES level. Percent spliced-in values (PSI) refer to the proportion of alternative isoforms at a splice site, where a PSI value of 1 or 0 indicates that only one of the two alternative isoforms at a splice site, and a value of 0.5 indicates equal expression of both isoforms. Numbers on each cluster represent the bootstrap probability values. C the average percent spliced-in value (PSI) of all isoforms across all fighting groups. Significance values were calculated using a Wilcoxon’s singled-rank test. [file 12864_2022_8609_MOESM11_ESM.png]
